# Flourishing in the Workplace: A One-Year Prospective Study on the Effects of Perceived Organizational Support and Psychological Capital

**DOI:** 10.3390/ijerph19020922

**Published:** 2022-01-14

**Authors:** Henry C. Y. Ho, Ying Chuen Chan

**Affiliations:** 1 Department of Psychology and Centre for Psychosocial Health, The Education University of Hong Kong, Hong Kong; 2 Department of Psychology, The Education University of Hong Kong, Hong Kong; yingchuen@eduhk.hk

**Keywords:** perceived organizational support, psychological capital, flourishing, PERMA, well-being

## Abstract

People working in urban areas often experience various work-related stressors, such as long working hours, high work pressure, and work-life interference, which can lead to severe mental and physical consequences. Identification of the protective factors that enable employees to flourish and thrive is especially important. The present study aims to identify the organizational and personal resources that contribute to employee flourishing. Adopting the conservation of resources theory and organizational support theory, it was hypothesized that perceived organizational support (POS) would promote employee flourishing through increasing psychological capital (PsyCap). A prospective study was conducted on a sample of 400 working adults from the social and personal services industry in Hong Kong. Data were collected at baseline, three months, and one year. Both Diener’s composite model of flourishing and Seligman’s PERMA model of flourishing were tested. Structural equation modeling showed that PsyCap at three months significantly mediated the effects of POS at baseline on flourishing and all dimensions of PERMA (positive emotion, engagement, relationships, meaning, accomplishments) at one year. Findings suggest that PsyCap underlies the process through which POS influences flourishing, whereby the promotion of positive psychological resources in a nurturing and supportive organization contributes to psychosocial functioning in the long run.

## 1. Introduction

People working in urban areas often experience various work-related stress, including long working hours, high work pressure, and work-life interference. The World Health Organization (WHO) recently included occupational burnout, a syndrome resulting from chronic work stress, in the International Classification of Diseases (ICD-11) as an occupational phenomenon that may influence health status [1]. Hong Kong, in particular, has received growing concern for occupational health as it is ranked as the most overworked city in the world [2]. Local surveys showed that over 40% of working adults reported high levels of job stress and burnout, over 40% suffered from anxiety, and 20% suffered from depression [3,4]. Yet, research suggests that employee well-being is a key contributor to job performance, absenteeism, and turnover [5]. The pursuit of a happy and satisfied workforce is an important goal not only as an end in itself but also as a means to employers’ desired productivity level. Therefore, identification of the protective factors that enable employees to flourish and thrive is a timely and vital endeavor.

According to Diener et al. [6], flourishing refers to psychosocial functioning, which is achieved by meeting the human psychological need for relationships, respect, self-esteem, competence, meaning, and purpose in life. They developed the Flourishing Scale as a summary measure of this construct [6]. Seligman [7] proposed the PERMA model, which advocates that flourishing is derived from five pillars of well-being: *P*ositive emotion, *E*ngagement, *R*elationships, *M*eaning, and *A*ccomplishments. It emphasizes that PERMA are the building blocks for profound fulfillment in life. The PERMA-Profiler was developed as a multidimensional measure to capture the five dimensions [8]. The two models agree that flourishing involves maximizing one’s potentials and living to the fullest to achieve optimal psychosocial functioning. While the literature on occupational health has predominantly focused on work-related well-being (e.g., job satisfaction) and ill-being (e.g., occupational burnout), research on employee flourishing is scarce. This study aims to identify the organizational and personal resources that contribute to employee flourishing and clarify the mechanism of this process.

### 1.1. Theoretical Background of Psychological Capital

This study adopted positive organizational behavior (POB) as the guiding framework to examine the determinants of employee flourishing. POB applies the developments in positive psychology to the workplace with emphasis on human resource strengths and psychological resources that are positive and desirable for flourishing and thriving employees [9]. Central to POB are efforts to integrate organization-centered focus on performance improvement with employee-centered focus on well-being and health promotion by considering both pursuits as equally valuable goals that yield positive work outcomes [10]. This movement involves identification and application of positively-oriented psychological constructs that are theoretically and psychometrically sound, empirically supported, and open to development in the workplace. The concept of psychological capital (PsyCap) was derived from this endeavor [11]. PsyCap is a higher-order construct that encompasses *H*ope, *E*fficacy, *R*esilience, and *O*ptimism (HERO). Individuals who possess a high level of PsyCap are devoted to pursue meaningful goals and determined to generate alternative pathways to achieve success (hope); confident about their abilities to execute the necessary courses of action in a successful way (efficacy); able to overcome and bounce back from adversities, setbacks, and failures (resilience); and likely to hold a generalized positive outlook by attributing events in a favorable light (optimism). PsyCap has received psychometric support, including construct, convergent and criterion validity, internal reliability, and incremental validity beyond its individual components [12].

Meta-analysis of PsyCap revealed its salutary effects on a wide range of employee outcomes, including psychological well-being, work-related well-being, and job performance [13]. A cross-sectional survey of employees from a retail company showed that PsyCap was positively associated with work engagement and negatively associated with psychological distress [14]. A cross-sectional survey of police officers revealed that PsyCap was positively associated with job satisfaction and negatively associated with stress symptoms [15]. A two-wave prospective study of social workers indicated that PsyCap can predict job satisfaction, job-related affect, and stress symptoms three months later [16]. However, whether PsyCap is associated with employee flourishing over time remains largely unknown.

### 1.2. Organizational and Personal Resources for Flourishing

According to the conservation of resources theory (COR), people are driven by the primary goal to obtain, preserve, and protect resources that they centrally value [17]. Key resources are conditions (e.g., social support), personal characteristics (e.g., self-efficacy), and energies (e.g., effort). Stress increases when resources are threatened or depleted. An important assumption of COR is that people have to invest resources to better cope and deal with stressful situations so that negative outcomes are prevented, and desirable goals are achieved. Therefore, PsyCap is an important personal resource for confronting challenges, resolving work problems, and promoting employee well-being [16,18,19]. COR also states that a reservoir of resources has to be acquired and accumulated to prepare for future investments. Personal resources can be cultivated together as resource caravans when the environment is nurturing and supportive [20]. Individuals working in a supportive organization are likely to be hopeful, efficacious, resilient, and optimistic. This resource caravan passageway is expected to lead to employee flourishing in the long run.

The organizational support theory holds that perceived organizational support (POS), which refers to employees’ perception that the organization cares about their well-being, recognizes their contributions, and makes an effort to fulfil their needs, is integral to their well-being [21]. POS exerts impact on work outcomes through self-enhancement and social exchange processes [22]. For self-enhancement, POS helps employees fulfill psychosocial and emotional needs, which could in turn lead to employee flourishing. Specifically, POS instills the notion that the organization values employees’ goals, which fulfills the need to be hopeful; POS conveys that the organization takes pride in employees’ accomplishments, which fulfills the need to be efficacious; POS indicates to employees that the organization is available to provide help when needed, which fulfills the need to be resilient; POS conveys the understanding that the organization considers employees’ best interest, which fulfills the need to be optimistic. For social exchange, POS is a valuable condition resource that develops a sense of obligation, gratitude, and reward-expectancy so employees are likely to reciprocate by investing their personal resources (e.g., PsyCap) to fulfill job demands [22]. In line with COR, meaningful investment of resources can in turn lead to employee flourishing because it protects against resource loss and gains resources that are important to overall well-being [17]. Studies showed that POS is associated with job satisfaction, job-related affect, and subjective well-being and reduced stress and burnout [21]. POS also has an indirect effect on depressive symptoms through PsyCap [23]. Based on the theoretical assertions and empirical evidence, the present study proposes that POS can increase PsyCap and in turn promote employee flourishing over time.

### 1.3. Present Study

By synthesizing three different literatures, an integrated model is generated and examined to provide theoretical insight on the resource caravan passageways of the conservation of resources theory, the self-enhancement and social exchange processes of the organizational support theory, and the common determinants of Diener’s et al. [6] and Seligman’s [7] models of flourishing. A meta-analysis showed that 88% of employee well-being research is cross-sectional, which has led to inflated correlations between variables [24]. A three-wave prospective design was adopted in this study to account for time sequence of the relationships between variables in order to produce more rigorous inferences [25]. It was hypothesized that (1) POS at Time 1 (T1) is positively associated with flourishing at Time 3 (T3); (2) POS at T1 is positively associated with PsyCap at Time 2 (T2); (3) PsyCap at T2 is positively associated with flourishing at T3; and (4) the association between POS at T1 and flourishing at T3 is mediated by PsyCap at T2 such that POS has a positive indirect effect on flourishing through PsyCap.

## 2. Methods

### 2.1. Participants and Procedures

A three-wave prospective study with a one-year duration was conducted. Data were collected at baseline (T1), three months (T2), and one year (T3). This time lag is appropriate for maintaining acceptable retention rates and managing attrition of subjects in follow-up assessments. As recommended for best practice, including three waves strengthens statistical modeling for testing directional inferences [26].

The study sample consisted of full-time workers from the social and personal services industry, which is one of the largest economic activities in the human services sector in Hong Kong [27]. They were recruited with the assistance of a local trade union that served these employees. Targeting the labor force of a specific industry controls for potential confounding influences from job nature, physical environment, and industry-specific policies on employee well-being. Participants received a link via email to complete an online survey at the three time-points. They received a HKD100 supermarket coupon as incentive for completing a survey at each time-point. This study was approved by the Human Research Ethics Committee of the university (Ref. no. 2019-2020-0249).

In addition to identification through the trade union, filter questions were included in the survey to ensure that the participants met the recruitment criteria of working full-time in the target industry. Among the 400 eligible participants recruited at T1, 316 (79%) of them responded at T2, and 303 (76%) of them responded at T3. The original sample size was determined using power analysis for structural equation modeling [28] with a statistical power of 0.95, alpha of 0.05, and medium effect size of 0.30. Participants were predominantly female (76.7%), aged between 30 and 39 years (53.3%), held a bachelor (38.8%) or master’s degree (45.7%), and worked in the industry for 11.3 years (*SD* = 7.15).

### 2.2. Measures

Perceived organizational support. The 16-item Survey of Perceived Organizational Support [29] was used to measure the extent to which the organization is committed to support its employees, values their continued contribution, and has concern about their well-being. Responses are given on a 7-point scale (1 = strongly disagree; 7 = strongly agree), with a higher total score indicating higher level of POS. An example of the scale is “The organization really cares about my well-being”. It has been validated in multiple languages and widely adopted in various populations, including Chinese [30]. It has yielded a high internal reliability of 0.95 in the present study.

Psychological capital. The 24-item Psychological Capital Questionnaire [12] was used to measure PsyCap as a multidimensional higher-order construct. The items represent hope, efficacy, resilience, and optimism, which are rated on a 6-point scale (1 = strongly disagree; 6 = strongly agree). A higher total score indicates a higher level of PsyCap. An example of the scale is “I always look on the bright side of things regarding my job”. It has been translated and validated in various languages and is widely used in Chinese populations [31]. It has yielded a high internal reliability of 0.95 in the present study.

Flourishing. The 8-item Flourishing Scale [6] was used as a composite measure of psychosocial well-being. The scale encompasses the most important aspects of human functioning, including relationships, respect, self-esteem, competence, meaning, and purpose in life. The items are rated on a 7-point scale (1 = strongly disagree; 7 = strongly agree), with a higher total score indicating a higher level of flourishing. An example of the scale is “I lead a purposeful and meaningful life”. It has been translated and validated in various languages for different populations, including Chinese [32]. It has yielded a high internal reliability of 0.90 in the present study.

PERMA. The 15-item PERMA-Profiler [8] was used as a multidimensional measure of the five pillars of well-being, including positive emotion, engagement, relationships, meaning, and accomplishments. Each dimension is indicated by three items that are rated on an 11-point scale (0 = never; 11 = always). A higher subscale score indicates higher level of a specific aspect of psychosocial well-being. An example of the scale is “How much of the time do you feel you are making progress towards accomplishing your goals?” It has been translated in multiple languages, including Chinese, and has demonstrated content, convergent, and divergent validity [8]. It has yielded an acceptable to high internal reliability for positive emotion (0.91), engagement (0.68), relationships (0.88), meaning (0.94), and accomplishments (0.83) in the present study.

### 2.3. Data Analysis

Data were analyzed using SPSS and Mplus. Missing data are common in studies with multiple time-points, but existing research often adopts complete case analysis, which could lead to biased parameter estimates and substantial loss of power [33]. Multiple imputation (MI) produces greater efficiency and less biased estimates than traditional approaches [34]. MI was carried out with fully conditional specification using the Markov chain Monte Carlo algorithm, which is effective even on datasets with complex patterns of missingness [35]. Structural equation modeling was conducted to examine the direct and indirect associations between variables. PsyCap was analyzed as a higher-order construct encompassing the first-order latent variables of hope, efficacy, resilience, and optimism [12]. As suggested by Rogers and Schmitt [36], each latent variable was indicated by three parcels of manifest variables produced by the factorial algorithm, which addresses item-level non-normality issues, number of sources of sampling error, cross-loadings between different factors, and correlated residuals. Confirmatory factor analysis was conducted to assess whether the data fitted a measurement model consisting of the latent constructs. Prospective mediation analysis was conducted via a structural model with POS at T1 as predictor, PsyCap at T2 as mediator, and flourishing at T3 as outcome. Both Diener’s et al. [6] composite model of flourishing and Seligman’s [7] PERMA model of flourishing were tested to cross-validate the hypotheses. Age, sex, education level, and work experience were accounted for in the analysis. Indirect effects were examined using sobel test [37]. Goodness-of-fit indices were used to appraise model fit: CFI and TLI ≥ 0.90 and RMSEA and SRMR ≤ 0.08 [38].

## 3. Results

### 3.1. Inter-Correlations

Pearson correlations are presented in Table 1. POS at T1 was positively correlated with PsyCap at T2 (*r* = 0.32, *p* < 0.001), flourishing at T3 (*r* = 0.27, *p* < 0.001), and all dimensions of PERMA at T3 (*r* = 0.21–0.30, *p* < 0.001). PsyCap at T2 was positively correlated with flourishing (*r* = 0.43, *p* < 0.001) and all dimensions of PERMA at T3 (*r* = 0.36–0.49, *p* < 0.001).

### 3.2. Measurement Model

The measurement models showed that all of the standardized factor loadings were statistically significant (*p* < 0.001). The factor loadings for POS ranged from 0.89 to 0.95. The factor loadings for the first-order latent variables of PsyCap ranged from 0.79 to 0.83 for hope, 0.73 to 0.81 for efficacy, 0.61 to 0.71 for resilience, and 0.52 to 0.83 for optimism. For the higher-order model of PsyCap, they ranged from 0.79 to 0.97. For flourishing, they ranged from 0.72 to 0.90. For the five dimensions of PERMA, they ranged from 0.81 to 0.84 for positive emotions, 0.44 to 0.80 for engagement, 0.75 to 0.85 for relationships, 0.84 to 0.89 for meaning, and 0.60 to 0.82 for accomplishments. The goodness-of-fit of the measurement models (Table 2) were excellent for the flourishing model (χ^2^ (128) = 151.42 (*p* = 0.08), CFI = 0.99, TLI = 0.99, RMSEA = 0.02, SRMR = 0.04) and the PERMA model (χ^2^ (380) = 356.45 (*p* = 0.80), CFI = 1.00, TLI = 1.00, RMSEA = 0.00, SRMR = 0.04). The results provided evidence of construct validity for POS, PsyCap, flourishing, and PERMA.

### 3.3. Structural Model

**Figure 1:**
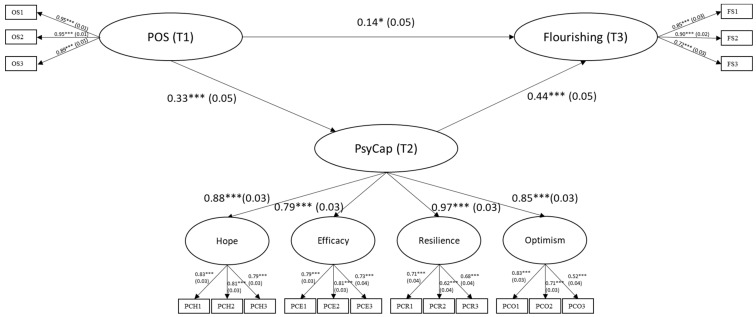
Structural Equation Model from Perceived Organizational Support to Psychological Capital to Flourishing. (Note. Standardized factor loadings and standardized path coefficients are shown. Measurement errors are not shown for clarity. * *p* < 0.05; *** *p* < 0.001).

Results from the flourishing model are illustrated in Figure 1. The direct effects of POS at T1 on PsyCap at T2 and flourishing at T3 were positive and significant (β = 0.33, *p* < 0.001; β = 0.14, *p* = 0.01, respectively). PsyCap at T2 also had a positive direct effect on flourishing at T3 (β = 0.44, *p* < 0.001). POS at T1 had a significant indirect effect through PsyCap at T2 on flourishing at T3 (Table 3). The goodness-of-fit of the structural model was excellent: χ^2^ (192) = 212.07 (*p* = 0.15), CFI = 0.99, TLI = 0.99, RMSEA = 0.02, SRMR = 0.04 (Table 2).

**Figure 2:**
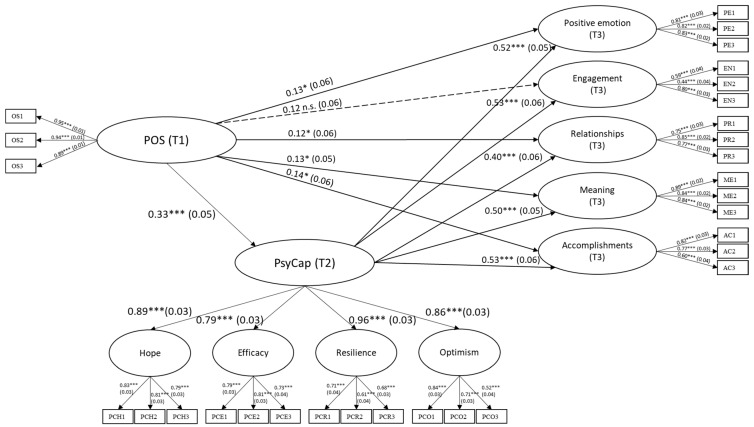
Structural Equation Model from Perceived Organizational Support to Psychological Capital to PERMA. (Note. Standardized factor loadings and standardized path coefficients are shown. Measurement errors are not shown for clarity. * *p* < 0.05; *** *p* < 0.001).

Results from the PERMA model are illustrated in Figure 2. The direct effects of POS at T1 on PsyCap at T2 (β = 0.33, *p* < 0.001), as well as positive emotion (β = 0.13, *p* = 0.02), relationships (β = 0.12, *p* = 0.04), meaning (β = 0.13, *p* = 0.01), and accomplishments (β = 0.14, *p* = 0.01) at T3 were positive and significant. The direct effect of POS at T1 on engagement at T3 was non-significant (β = 0.12, *p* = 0.06). The direct effects of PsyCap at T2 on positive emotion (β = 0.52, *p* < 0.001), engagement (β = 0.53, *p* < 0.001), relationships (β = 0.40, *p* < 0.001), meaning (β = 0.50, *p* < 0.001), and accomplishments (β = 0.53, *p* < 0.001) at T3 were positive and significant. POS at T1 had significant indirect effects through PsyCap at T2 on all dimensions of PERMA at T3 (Table 3). The goodness-of-fit of the structural model was excellent: χ^2^ (476) = 431.14 (*p* = 0.93), CFI = 1.00, TLI = 1.00, RMSEA = 0.00, SRMR = 0.04 (Table 2).

## 4. Discussion

**Figure 3:**
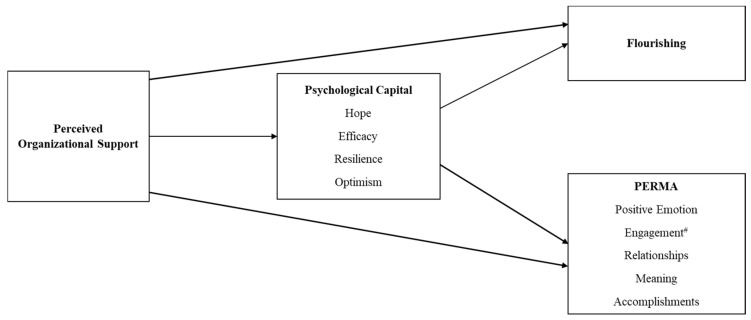
Consolidated Model of the Relationships between Perceived Organizational Support, Psychological Capital, Flourishing, and PERMA. (Note. ^#^ Relationship between perceived organizational support and engagement is non-significant. All other relationships are statistically significant).

This is the first study to adopt a one-year three-wave prospective design to investigate the long-term associations between POS and employee flourishing, as mediated by PsyCap. Largely consistent with H1, the results showed that POS at baseline had direct positive effects on flourishing and four dimensions of PERMA one year later. Consistent with H2, POS at baseline had direct positive effects on PsyCap three months later. Consistent with H3 and H4, PsyCap at three months had direct positive effects on flourishing and all dimensions of PERMA at one year and served as a mediator of the relationships between POS and psychosocial well-being. A consolidated model of the results is illustrated in Figure 3.

In support of the organizational support theory [21], our study demonstrated that POS is an important contributor to employee flourishing. Employees who perceived that the organization values their contribution, cares about their well-being, and commits to provide support were more likely to experience flourishing in the long run. They were more likely to feel contentment and joy, establish and maintain positive relationships with others, have a sense of purpose and meaning in life, and work towards or achieve goals that they have set for themselves. These findings are in line with the existing literature on the positive associations between POS and work-specific outcomes, including job-related affect [39], work relationships [40,41], meaningful work [42], and work accomplishments [43,44]. Our findings suggest that the salutary effects of POS extend beyond the work domain to fulfil universal facets of psychosocial and emotional needs of workers. Future research are needed to investigate the benefits of organizational resources beyond the work domain, especially to identify the work-related factors that contribute to employee growth and flourishing. For example, organizational justice [45] may be another characteristic of work that can satisfy employees’ need for engagement, meaning, and accomplishments. An organizational culture that emphasizes staff-centeredness and teamwork [46] may also promote the psychosocial functioning of employees.

The mediating role of PsyCap on the associations between POS and employee flourishing was consistent with the propositions of the conservation of resources theory [17]. This finding suggests that PsyCap underlies the process through which POS influences flourishing, whereby the promotion of positive psychological resources in a nurturing and supportive organization contributes to psychosocial functioning in the long run. It applies to the composite conceptualization of flourishing as well as the unique dimensions of PERMA, suggesting that resourceful employees not only feel happy and satisfied with life in general, but they also proactively pursue each of the building blocks of well-being for profound fulfillment in life. This is largely consistent with COR, which states that supportive organizations cultivate personal resources together as resource caravans and that individuals have to invest these resources (i.e., work on the building blocks of well-being) to achieve desirable outcomes [20]. The present study sheds light on the directional influence of POS and PsyCap over time and the benefits of being hopeful, efficacious, resilient, and optimistic for employee flourishing. While the ongoing debate about the conceptualization of flourishing is outside the scope of this study [47,48], our results provided evidence of convergent validity, whereby POS and PsyCap are common predictors of both overall well-being and building blocks of well-being.

### Limitations

The study should be considered with the following limitations. First, the findings might be susceptible to self-selection bias since the working adults volunteered to participate in this study. Due to self-selection, the sample characteristics may be different from people who chose not to participate [49]. For example, someone who is dealing with an unsupportive employer and therefore has a low reserve of psychological resources might refrain from spending time and effort in a commitment-intensive study. Second, the participants were employees in the social and personal services industry, which may limit the generalizability of the results to workers from manual labor industries. Interpretation of the study findings should be considered in the human services sector. Third, since questionnaires were used as the data collection method, there may be social desirability bias. Nevertheless, self-report is valid and reliable in well-being research because the nature of the research question is fundamentally subjective [50]. Self-reported measures of quality of life are instrumental for both theory building and program enhancement [51]. Finally, although time lags of three months and one year were used to separate the assessment time of the variables to account for the temporal order of relationships, causal inferences cannot be made definitively. Chronological observations were limited to the specific time frame of the study. Nevertheless, this prospective study has a longer time lag than many similar studies with a working sample, e.g., [31,52].

## 5. Conclusions

The findings of this study provide important implications at the organizational level and at a personal level. Since POS is an important contributor for personal resources and well-being among employees, organizations are suggested to implement employee-oriented practices that value the contributions of employees, acknowledge their accomplishments, recognize their personal goals and values, encourage them to voice out their opinions and concerns, provide assistance in times of need, show concern for their well-being, and foster a sense of belonging at work. Based on the POS literature, it can be achieved through fair resource distribution, favorable performance-reward systems, job security, job autonomy, and work–life balance practices [53]. While prior research suggests that social capital in organizations can promote effective management strategies and in turn better teamwork and work engagement [54,55], the present study showed that it could ultimately promote flourishing.

On a personal level, training programs can be designed and developed to promote PsyCap among working adults since it is malleable to change and development [56]. To instill hope among employees, the training involves identifying personal goals and multiple pathways to achieve them. Obstacles are identified, and solutions are planned out. Through breaking down the overarching goal into smaller steps, efficacy is developed as individual goals are attained. Resilience can be increased through developing alternative pathways when obstacles are encountered in order to bounce back from setbacks. For optimism, employees are trained to heighten their awareness of pessimistic thoughts and actively challenge them with realistic expectations about the future. Through isolating negative distortions and practicing being realistically optimistic, positive expectations for success is increased. Prior research showed that PsyCap training is effective for cultivating hope, efficacy, resilience, and optimism as well as promoting life satisfaction, work engagement, and job performance [57]. The present study suggests that employee flourishing could be a distal outcome of PsyCap training programs and therefore should be further developed and put into practice.

## Figures and Tables

**Table 1:** Zero-order correlations between variables.

	1	2	3	4	5	6	7
POS T1							
2. PsyCap T2	0.32 ***						
3. Flourishing T3	0.27 ***	0.43 ***					
4. Positive Emotion T3	0.30 ***	0.47 ***	0.73 ***				
5. Engagement T3	0.21 ***	0.39 ***	0.65 ***	0.66 ***			
6. Relationships T3	0.25 ***	0.36 ***	0.64 ***	0.69 ***	0.59 ***		
7. Meaning T3	0.29 ***	0.47 ***	0.78 ***	0.76 ***	0.68 ***	0.73 ***	
8. Accomplishments T3	0.29 ***	0.49 ***	0.62 ***	0.67 ***	0.70 ***	0.52 ***	0.66 ***

Note. POS, perceived organizational support; PsyCap, Psychological Capital; T1, baseline; T2, 3-month follow-up; T3, 1-year follow-up. *** *p* < 0.001.

**Table 2:** Goodness-of-fit Indices of the Measurement Model and Structural Model.

Fit Index	Measurement Model		Structural Model	
	Flourishing	PERMA	Flourishing	PERMA
χ^2^	151.42	356.45	212.07	431.14
DF	128	380	192	476
*p*	0.08	0.80	0.15	0.93
CFI	0.99	1.00	0.99	1.00
TLI	0.99	1.00	0.99	1.00
RMSEA	0.02	0.00	0.02	0.00
SRMR	0.04	0.04	0.04	0.04

Note. χ^2^, chi-square; DF, degrees of freedom; *p*, *p*-value; CFI, comparative fit index; TLI, Tucker–Lewis index; RMSEA, root mean square error of approximation; SRMR, standardized root mean square residual.

**Table 3:** Prospective Mediation Analysis from Perceived Organizational Support to Psychological Capital to Flourishing and PERMA.

Model	Independent Variable	Dependent Variable	Direct Effect		Indirect Effect (PsyCap as Mediator)		
			β	*p*	β	*p*	(95% CI)
Model 1	POS T1	Flourishing T3	0.14	0.01	0.15	<0.001	(0.09, 0.20)
	POS T1	PsyCap T2	0.33	<0.001			
	PsyCap T2	Flourishing T3	0.44	<0.001			
Model 2	POS T1	Positive Emotion T3	0.13	0.02	0.17	<0.001	(0.11, 0.24)
	POS T1	Engagement T3	0.12	0.06	0.18	<0.001	(0.11, 0.25)
	POS T1	Relationships T3	0.12	0.04	0.14	<0.001	(0.08, 0.19)
	POS T1	Meaning T3	0.13	0.01	0.17	<0.001	(0.11, 0.23)
	POS T1	Accomplishments T3	0.14	0.01	0.18	<0.001	(0.11, 0.24)
	POS T1	PsyCap T2	0.33	<0.001			
	PsyCap T2	Positive Emotion T3	0.52	<0.001			
	PsyCap T2	Engagement T3	0.53	<0.001			
	PsyCap T2	Relationships T3	0.40	<0.001			
	PsyCap T2	Meaning T3	0.50	<0.001			
	PsyCap T2	Accomplishments T3	0.53	<0.001			

Note. Model 1: Diener’s composite model of flourishing as dependent variable; Model 2: Seligman’s PERMA model of flourishing as dependent variables. β, beta; *p*, *p*-value; CI, confidence interval; POS, perceived organizational support; PsyCap, Psychological Capital.; T1, baseline; T2, 3-month follow-up; T3, 1-year follow-up.

## Data Availability

The data that support the findings of this study are available from the corresponding author upon reasonable request.
